# Recent advances in acoustofluidic separation technology in biology

**DOI:** 10.1038/s41378-022-00435-6

**Published:** 2022-09-01

**Authors:** Yanping Fan, Xuan Wang, Jiaqi Ren, Francis Lin, Jiandong Wu

**Affiliations:** 1 School of Optical-Electrical and Computer Engineering, University of Shanghai for Science and Technology, Shanghai, 200093 China; 2 Institute of Biomedical and Health Engineering, Shenzhen Institute of Advanced Technology, Chinese Academy of Sciences, Shenzhen, 518055 China; 3 Department of Physics and Astronomy, University of Manitoba, Winnipeg, MB R3T 2N2 Canada

**Keywords:** Engineering, Nanoscience and technology, Materials science

## Abstract

Acoustofluidic separation of cells and particles is an emerging technology that integrates acoustics and microfluidics. In the last decade, this technology has attracted significant attention due to its biocompatible, contactless, and label-free nature. It has been widely validated in the separation of cells and submicron bioparticles and shows great potential in different biological and biomedical applications. This review first introduces the theories and mechanisms of acoustofluidic separation. Then, various applications of this technology in the separation of biological particles such as cells, viruses, biomolecules, and exosomes are summarized. Finally, we discuss the challenges and future prospects of this field.

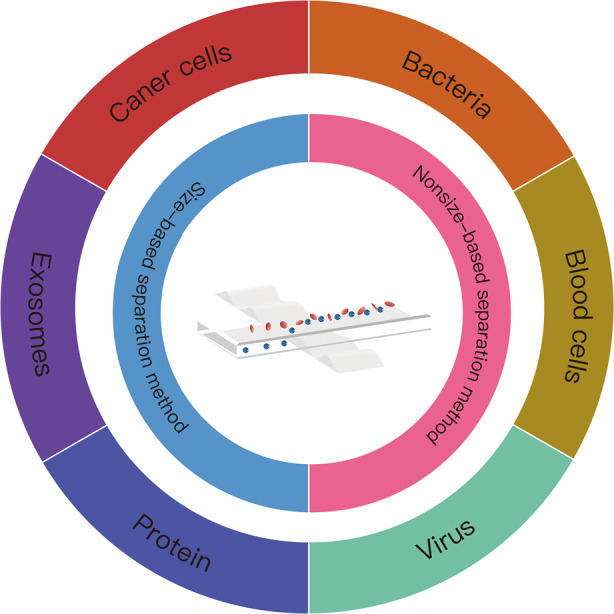

## Introduction

The separation of specific substances from mixtures has been applied in many fields, such as biological research^1^, chemical analysis^2^, and disease diagnosis^3^. Over the past decades, attention has been drawn to the separation of cells and bioparticles for different biological and biomedical applications. The diagnosis of certain diseases is determined by detecting relevant pathogens or cells, and the effective separation of these particles is an important basis for accurate detection. For a long time, several traditional separation technologies have been applied to accomplish this task^4^. The density gradient centrifugal separation method applies a centrifugal force to separate by size and density^5^. However, it is time-consuming and can lead to substantial cell loss and alter cell functions. The immuno-magnetic bead separation method is another widely used separation technology that separates cells based on the antigen-antibody reaction^6^. However, the lack of specific antibodies for certain cells makes this method not universal. New cell or particle separation methods are needed to overcome the limitations of these traditional technologies. Microfluidic devices have been recognized as a promising method to accomplish this task^7^. These devices can be roughly classified into two categories according to separation principle: passive methods and active methods. Methods that utilize a specially designed channel or fluid flow to change the trajectory of particles are referred to as passive separation methods, which include microstructural filtration, hydraulic, deterministic lateral migration, and inertia-based separation methods^8^. Active separation methods use an additional field (e.g., electrical, magnetic, acoustic, and optical fields) to generate a force that is exerted on particles and cells to achieve separation^9^. In general, passive methods require simpler equipment, while active methods achieve higher separation efficiency.

Acoustofluidics is an emerging technology that integrates acoustic waves with microfluidic systems to manipulate fluids and particles in microstructures. The concept of acoustofluidics was first proposed by Henrik Bruus in 2008^10^. He also made significant contributions to the theories of acoustofluidic separation over the following years^11–13^. The acoustofluidic method can separate different particles based on their physical properties, such as size^14^, density^15^ and compressibility^16^. Due to its label-free^17^, contactless^18^, and biocompatible features^19^, the acoustofluidic method has been recognized as a promising technology for the separation and manipulation of cells and bioparticles. One advantage of acoustofluidic technology is that it allows separation in a variety of fluid media due to the good penetrability of sound waves, whereas optical force-based methods are often affected by fluid properties such as transparency and turbidity. In addition, a wide range of acoustic frequencies (1 kHz–500 MHz) provides flexibility for different separation applications. Several previously reported reviews have nicely summarized acoustofluidic technologies and their bioapplications^4,20–23^. Instead of covering a broad range of applications in the biomedical/bioanalytical fields, this review mainly focuses on the acoustofluidic separation of cells and bionanoparticles, which can provide more relevant information for readers interested in this specific field. Compared with those reviews that also focus on particle separation, this paper was intended to provide more systematic and complete knowledge about the theories and mechanisms of separation, which may help readers, especially those new to the field, gain a more comprehensive understanding of this technology. Furthermore, this paper was updated with the latest literature in this area. In general, this review first introduces the basics of the theory and mechanism of acoustofluidic separation, then highlights the recent progress of the technology in biological applications, and finally discusses the challenges and perspectives of this field.

## Theories of acoustofluidic separation

In this section, we will introduce the type of acoustic waves used, the principle of acoustic excitation, and several parameters that play important roles in determining the migration of the sorting targets, including Rayleigh angle, Stokes force, and acoustic radiation force (ARF).

### Acoustic waves

Acoustic waves are mechanical waves generated by the high-frequency vibration of piezoelectric materials (e.g., lithium niobate, lithium tantalite, quartz) when alternating current (AC) electrical signals act on them^24^. Depending on whether the entire body or just the surface of the material vibrates, acoustic waves can be divided into bulk acoustic waves (BAWs) and surface acoustic waves (SAWs). Additionally, acoustic waves are also distinguished as traveling waves or standing waves in the field of acoustics. Traveling waves are unidirectional waves with regular propagation, whereas standing waves are composite waves that transmit bilaterally.

**Fig. 1:**
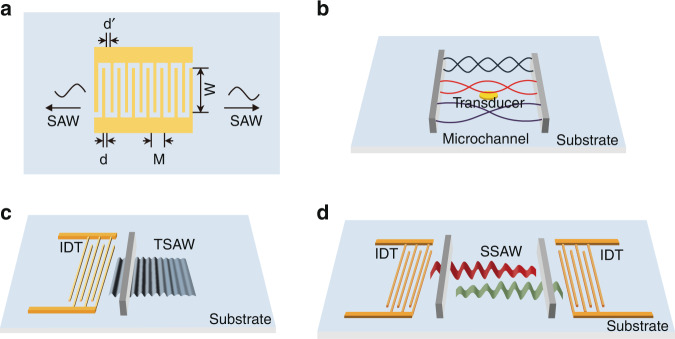
Different types of acoustic waves. **a** Schematic diagram of a surface acoustic wave generator; *d* represents the width of the finger bar, *d’* represents the width between the fingers, *M* represents the length of the periodic section and *W* represents the acoustic aperture; **b** schematic diagram of bulk acoustic waves; **c** schematic diagram of traveling surface acoustic waves; **d** schematic diagram of standing surface acoustic waves.

BAWs are standing waves that propagate in the microchannel, which defines the resonance chamber or cavity (Fig. 1b)^20^. The acoustic waves travel into the microchannel through the solid–liquid interface after the piezoelectric material is activated and resonates within the channel when the channel width is an integer multiple of half-wavelength. The reflection of acoustic waves from the channel wall results in the emergence of BAWs. Because the formation of BAWs depends on reflection from the channel wall, soft polymer materials, such as polydimethylsiloxane (PDMS), are not suitable for channel materials, whereas materials with excellent acoustic properties, such as silicon and glass, are suitable for channel fabrication. The acoustic impedance of the substrate and the quality factor of the resonator are important for BAW propagation^25^. Both the inhomogeneity of the acoustic impedance of the piezoelectric substrate and the low quality factor of the resonator can cause significant attenuation of BAWs.

SAWs are elastic waves that travel along the surface of piezoelectric materials. SAWs were discovered in 1885 by the British physicist Lord Rayleigh^26^. They can be further divided into Lamb waves, Love waves, surface transverse waves, horizontal shear waves, leaky surface acoustic waves, Rayleigh waves, and electroacoustic waves according to the difference in acoustic vibration modes and boundary conditions. The typical SAW generator (Fig. 1a) is composed of a piezoelectric material substrate and metal interdigital transducers (IDTs) depositing on it^27^. Due to the inverse piezoelectric effect, the surface of the piezoelectric substrate undergoes subtle mechanical deformation when a sinusoidal AC signal is applied to the IDTs. As a result, the mechanical SAW is generated and propagates along the solid-air surface in the direction of deformation^28^. The wavelength of SAWs is dependent on the width and spacing between IDT fingers. In a uniform IDT where the width *d* is equal to the spacing *d’*, as shown in Fig. 1a, the wavelength can be calculated by *λ* = *4d*. The acoustic frequency is calculated using the following equation:

1 $${{{{f}}}} = v/\lambda$$

where *v* is the speed of sound in the piezoelectric substrate.

**Fig. 2:**
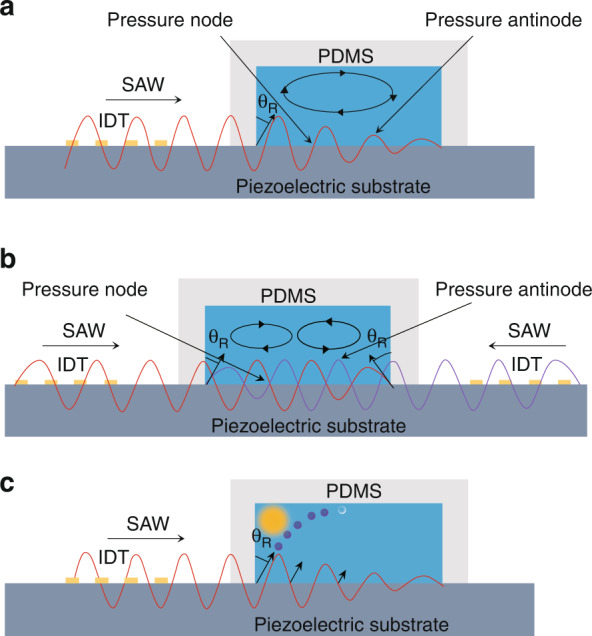
Schematic diagrams of different types of surface acoustic waves. **a** Acoustic streaming effect of traveling surface acoustic waves; **b** acoustic streaming effect of standing surface acoustic waves; **c** schematic diagram of the “anechoic corner effect”. The yellow area represents an anechoic domain where the streaming effects and acoustic radiation force are weak, so particles and cells are barely affected.

SAWs can be further categorized into traveling surface acoustic waves (TSAWs) and standing surface acoustic waves (SSAWs). TSAWs are generated by IDTs on one side (Figs. 1c, 2a). SSAWs are excited by two opposing IDTs, which create a pattern of minimum and maximum pressure regions called pressure nodes and pressure antinodes, respectively, within the channel through the interaction of acoustic waves and fluid (Figs. 1d, 2b).

### Rayleigh angle

The amplitude of SAWs decays exponentially as they transmit through the channel wall. The remaining acoustic waves continue to propagate along the substrate until the acoustic streaming coupling phenomenon occurs. As a result, “leakage surface acoustic waves” form in the microchannel^27^. Due to the difference in the viscosity between the fluid and substrate, the propagation speeds of SAWs in the two media are different. The acoustic waves refract at the fluid-solid interface and enter the fluid field with a refraction angle, which is defined as the Rayleigh angle^29^:

2 $$\theta _R = {\rm{arcsin}}\left( {C_1/Cs} \right)$$

where *C*_*1*_ and *C*_*s*_ are the acoustic wave velocities in the fluid and the piezoelectric substrate, respectively. The acoustic speed of the same material with different tangential directions varies according to the anisotropy of the piezoelectric materials. Therefore, the Rayleigh angle is related to the tangential direction of the piezoelectric substrate. Due to the Rayleigh angle, there is a unique phenomenon called the “anechoic corner effect”; that is, the extremely weak ARF at the upper corner of the microchannel can barely affect the particles there (Fig. 2c).

### Stokes force

Acoustic streaming is a stable flow driven by the absorption of acoustic oscillations as acoustic waves with high frequency and amplitude act on the fluid. The generation of acoustic streaming is a result of the viscous attenuation feature of the fluid^30^. The particles and cells in acoustic streaming are subjected to the resistance force of the fluid, which is called the Stokes force. The Stokes force (*F*_*d*_) can be calculated using the following formula:

3 $$F_d = 6\pi uR_pv$$

where *u*, *v*, and *R*_*p*_ denote the fluid viscosity, relative velocity between fluid and particles, and particle radius, respectively.

### Acoustic radiation force

In principle, the acoustic pressure gradient emerges due to nonlinear sound propagation in the fluid medium, which generates the ARF acting on the particles. There are two types of ARFs: the primary acoustic radiation force (PRF) and the secondary acoustic radiation force (SRF). In TSAWs, the trajectories of particles are determined by the interplay between the acoustic streaming-induced drag force and the ARF. The dominant force is determined by a dimensionless coefficient (*K*_*tr*_) that was introduced by Skowronek et al.^31^:

4 $$K_{tr} = 2\pi R_p/\lambda$$

where *R*_*p*_ is the radius of the particle and *λ* is the acoustic wavelength. When *K*_*tr*_ < 1, the acoustic streaming-induced drag force is dominant, and the particle moves in the acoustic streaming vortex. When *K*_*tr*_ > 1, the particle is mainly driven by the ARF and moves away from the IDT^32^. King derived the formula of a PRF acting on the particles in a TSAW as follows^33^:

5 $$F_{{\rm{PRF}}} = 2\pi \rho _l\left| A \right|^2\left( {kR_p} \right)^6\frac{{9 + 2\left( {1 - \lambda _p} \right)^2}}{{9\left( {2 + \lambda _p} \right)^2}}$$

6 $$\lambda _p = \frac{{\rho _l}}{{\rho _p}}$$

where *A* represents the complex amplitude of velocity potential; *k* represents the wavenumber of acoustic radiation; *R*_*p*_ represents particle radius; and *ρ*_*l*_ and *ρ*_*p*_ represent the density of the surrounding fluid and the particle, respectively.

In the SSAW field, PRF can be further divided into two components: the axial component (*F*_*a*_) and the transverse component (*F*_*t*_). *F*_*a*_ is calculated using the equation below^34^:

7 $${{{{F}}}}_a = - \left( {\frac{{\pi p_0^2V_p\beta _l}}{{2\lambda }}} \right)\phi \left( {{\upbeta }},{\uprho} \right){{{\mathrm{sin}}}}\left( {2{{{{kx}}}}} \right)$$

8 $$\phi \left( {{\upbeta }},{\uprho} \right) = \frac{{5\rho _p - 2\rho _l}}{{2\rho _p + \rho _l}} - \frac{{\beta _p}}{{\beta _l}}$$

where *ϕ* is the acoustic contrast factor; *P*_*0*_ is the acoustic pressure amplitude; *x* is the axial distance of the particle from the pressure node; *V*_*p*_ and *β*_*p*_ represent the volume and compressibility of the particle, respectively; and *β*_*l*_ denotes the compressibility of the fluid.

*F*_*a*_ can direct particles toward either pressure nodes or antinodes, which is determined by the acoustic contrast factor (*ϕ*). When *ϕ* > 0, particles move toward the pressure nodes; when *ϕ* < 0, particles move toward the pressure antinodes. When particles are pushed toward the nodal plane, the axial force is negligible, and the transverse pressure force becomes dominant. The equation of *F*_*t*_ is derived by the Whitworth function as follows^35^:

9 $${{{{F}}}}_t = 3d_p^3\frac{{\rho _{p - }\rho _l}}{{2\rho _p + \rho _l}}\nabla \left\langle {E_{{\rm{ac}}}} \right\rangle$$

where *∇* *<* *E*_ac_> represents the acoustic energy gradient, *<>* represents the time average, and *d*_*p*_ represents the distance between particles. The transverse pressure force pushes particles closer to each other. As the distance between the particles decreases, the force becomes weaker until the particles aggregate together at the pressure node or antinode.

Although the migration of a single particle is mainly affected by the PRF, the SRF becomes important when multiple cells or particles aggregate. As demonstrated by Saeidi et al., when the distance between particles is small, the trajectory of the particles is strongly affected by the SRF^36^. Silva and Bruus demonstrated that two particles in close proximity could either attract or repel each other in a direction that is perpendicular to the wave propagation within the Rayleigh limit when the acoustic wavelength is much greater than the particle size^37^. Hashmi et al. verified that whether the SRF acts as an attractive or repulsive force depends on the ratio of fluid density to particle density^38^. More recently, Mohapatra et al. showed that the interparticle ARF is proportional to the diameter of particles^39^. Wiser et al. derived the SRF equation of two particles with identical acoustic properties and radii in the Rayleigh limit^40^:

10 $$F_{\rm{SRF}}\left( x \right) = 4r^6\left[ {\frac{{\left( {\rho _p - \rho _f} \right)^2\left( {3cos^2\theta - 1} \right)}}{{6\rho _fd^4}}v^2\left( x \right) - \frac{{\omega ^2\rho \left( {k_p - k_f} \right)^2}}{{9d^2}}p^2\left( x \right)} \right]$$

where *v(x)* and *p(x)* represent the particle velocity and acoustic pressure, respectively, *θ* represents the angle between the centerline that connects two particles and the direction of acoustic wave propagation, *ω* is the angular frequency of the sound wave, and *d* is the distance between the centers of the two particles.

In the case of a particle close to a bubble, the SRF can be calculated using the following equation^41^:

11 $$F_{{\rm{SRF}}} = 4\pi \rho _l\frac{{\rho _l - \rho _p}}{{\rho _l + 2\rho _p}}\frac{{R_b^4R_P^3}}{{d^5}}\omega ^2\xi ^2$$

where *ρ*_*l*_ and *ρ*_*p*_ are the densities of fluid and particles, respectively; *d* is the distance between the particle and bubble; *ω* is the angular frequency; *ξ* is the amplitude of bubble oscillation; and *R*_*b*_ and *R*_*p*_ are the radius of the bubble and particle, respectively. The direction of the force depends on the values of *ρ*_*l*_ and *ρ*_*p*_, which determine whether the force is attractive or repulsive.

## Mechanisms of acoustofluidic separation

In addition to understanding the principles of acoustofluidic separation, researchers have also conducted experimental studies aimed at effectively separating particles or cells from mixtures. Various separation methods have been explored using different acoustofluidic devices. The basic mechanisms of these methods will be summarized in this section. Different types of acoustofluidic separation devices are listed in Table 1.

**Table 1:** Overview of different acoustofluidic separation devices.

Type of acoustic waves	Typical exciter	Features of devices	Ref.
Bulk acoustic waves	Piezoelectric material	Easy to fabricate, but difficult to manipulate pressure nodes and antinodes	^71,82^
Traveling surface acoustic waves	One IDT	Flexible to manipulate and generate acoustic streaming	^43,73^
Standing surface acoustic waves	A pair of opposing IDTs	Convenient control of pressure nodes and anti-nodes	^42,69,72^
Tilted-angle traveling surface acoustic waves	One IDT with tilted-angle along the channel	Increased migration distance of target particles	^60^
Tilted-angle standing surface acoustic waves	A pair of opposing IDTs with tilted-angle along the channel	Long distance migration and stable separation for multiple particles	^63,74^

### Separation methods based on size

Because the ARF acting on particles is proportional to the particle size, it is natural to use acoustic waves to separate particles of different sizes. Many acoustofluidic devices^42–45^ have been developed based on this separation principle. However, other factors, such as the structure and position of IDTs, can affect the outcomes in size-based separation.

**Fig. 3:**
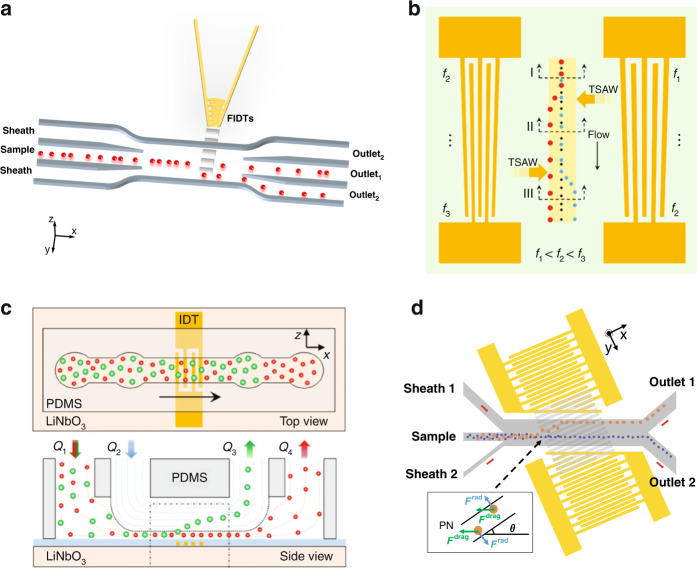
Size-based separation using different interdigital transducer designs and positions. **a** Focused interdigital transducers were placed beside the microchannel to generate high-energy-density traveling surface acoustic waves for particle separation. Reproduced from ref. ^49^ with permission from the Royal Society of Chemistry. **b** A pair of slanted interdigitated transducers placed on the two sides of the microchannel was activated by different frequency signals for particle separation. Reproduced from ref. ^53^ with permission from the American Chemistry Society. **c** An interdigital transducer placed under the microchannel was used to separate polystyrene particles of different sizes via vertical migration. Reproduced from ref. ^58^ with permission from Wiley Online Library. **d** A pair of tilted-angle interdigital transducers was used to enhance the cell deflection in the microchannel. Reproduced from ref. ^64^ with permission from the Institute of Electrical and Electronics Engineers.

The most common IDT structure is rectangular. Although widely used, this type of IDT is not suitable for applications that require high acoustic energy in a narrow domain. Therefore, some special IDT structures have been developed to meet the needs of different applications. One type of specially designed IDT is the focused interdigitated transducer (FIDT), which has arcuate IDT fingers, and one side of the FIDT is narrower than the other side^46^. A higher energy intensity and narrower beam width can be generated at the narrow side^47,48^. As a result, the force exerted on the particles and cells is stronger, and a higher sorting resolution can be realized. By using FIDTs, Collins et al. were able to generate an effective sorting region with a width of ~25 μm^49^. The submillisecond pulses generated at kHz rates allowed for the high-speed sorting of 2 μm particles from 1 μm particles (Fig. 3a). Later, the same group used FIDT to generate fluid vortices that extend over the entire channel width^50^. This design maximized the effect of acoustic streaming and was able to selectively capture 2 μm particles from a mixed suspension with 1 μm particles and capture human breast cancer cells from red blood cells (RBCs). Another type of specially designed IDT is the slanted interdigitated transducer (SIDT). In an SIDT, the distance between fingers on one side is narrower than that on the other side, resulting in a tapered shape of the entire structure. The change in finger spacing makes the optimum actuated frequency tunable over a wide range, which has the advantages of generating different amplitude profiles to facilitate the size-selective separation of particles or cells^51,52^. Destgeer et al. demonstrated the separation of polystyrene (PS) particles of three different sizes using a pair of SIDTs (Fig. 3b)^53^. By carefully designing the tapered shape, the left and right SIDTs could generate a range of different frequencies in different positions along the flow direction, forming the basis to manipulate different particles. In one set of experiments, the authors demonstrated that 5 µm particles deflected to the top-left anechoic corner upstream when *f*_*L*_ < *f*_*R*_, 4.2 µm particles deflected to the right side of the channel downstream when *f*_*L*_ > *f*_*R*_, 5 µm particles were unaffected in the anechoic corner, and the smallest 3 µm particles remained in the middle part of the channel due to the lower ARF exerted on them. Moreover, this device was shown to be useful for medium exchange application by alternatively moving the particles from the left to the right medium. Recently, Ji et al. developed an acoustofluidic device that integrates a spiral channel for sheathless focusing of the selected particles, an offset micropillar array for concentrating the particles on one side of the channel, and an SIDT for deflecting the target particles^54^. The experiment showed that this device achieved the separation of 20 µm particles with 92% purity and 100% efficiency.

In addition to the IDT structure, the position of the IDT can be adjusted to generate a special effect for particle separation. In classic SAW devices, IDTs are usually placed at a certain distance beside the microchannel. The separation is based on the horizontal displacement of particles. In some studies, IDTs are placed underneath the microchannel to allow vertical separation of particles and cells^55–57^. Using this strategy, Ahmed et al. developed a TSAW device to continuously separate particles of different sizes (Fig. 3c)^58^. This device took advantage of the vertical ARF component to push the selected particles upward in the microchannel. The horizontal ARF component was used to slow down the separated particles in the lateral direction, which also gave the particles more time for vertical migration and improves separation efficiency. They demonstrated the continuous separation of 4.8 µm PS particles from 2.0 and 3.2 µm ones at high efficiency (>99% purity and recovery). In typical acoustofluidic sorting devices, IDTs are usually placed parallel to the microchannel. This layout results in a maximum distance of one-quarter of the wavelength that the particles can deflect in SSAW devices^59^. These limitations can be addressed by putting the IDTs at an angle to the channel. This type of device includes tilted-angle traveling surface acoustic wave (taTSAW) devices and tilted-angle standing surface acoustic wave (taSSAW) devices. For taTSAWs, the IDT is placed on one side of the channel at a tilted angle to generate a traveling SAW that can be used to deflect the particles in the channel. Ahmed et al. developed a taTSAW device for sheathless focusing and separation of microparticles in a continuous flow^60^. A pair of IDTs was set below the channel at angles of 210° and 150° relative to the principal axis of the substrate wafer. The first IDT used a frequency of 194 MHz to push all particles to one side of the microchannel, while the second IDT separated 4.8 μm fluorescent PS particles from 3.2 μm particles using 136 MHz frequency with a purity > 99%. For the taSSAW, a pair of tilted-angle IDTs is patterned beside the microchannel to form a standing wave, which makes the pressure nodes or antinodes also not parallel to the channel^61,62^. Ding et al. developed a taSSAW device (with a 30° tilted angle) to separate PS beads with different diameters (2 and 10 µm) and achieved ~99% separation efficiency^63^. Multiple nodal lines crossed the channel because of the tilted angle of the IDT. Target particles deflected to one side of the channel by migrating toward the nodal lines, whereas nontarget particles were not affected because the ARF applied to them was weak. Recently, Wu et al. introduced a filled tilted-angle (FTA) SAW device, which effectively separated HeLa cancer cells from peripheral blood mononuclear cells (PBMCs)^64^. By filling the space adjacent to the microchannel with IDTs (Fig. 3d), the device exhibited a higher separation efficiency (32%) than the conventional system. At an input power of 4.5 W, the separation efficiencies for HeLa cells and PBMCs were approximately 90 and 25%, respectively. In most of the current SAW devices, the microchannel layers are irreversibly bonded to the IDT layer. While the devices in many biomedical applications should be single use, the cost of each test is high because of the high fabrication cost of IDTs. Detachable SAW devices have been developed to reduce this cost by reusing the IDT components. This requires the transmission of SAW energy from the IDT substrate to the microchannel superstrate. Different coupling agents and strategies have been explored^65–67^. Ma et al. designed a disposable TSAW-based separation device and successfully separated PS particles with sizes of 10 and 15 μm^68^. The microstructured pillar was used to bond the substrate and PDMS microchannel, which acts as the coupling agent and allows the detachment of the two components after use. Moreover, the unique connection mode effectively eliminated the “anechoic corner effect”, subjecting the entire channel to SAW, which also maximized the acoustic force for separation.

### Separation methods based on nonsize properties

**Fig. 4:**
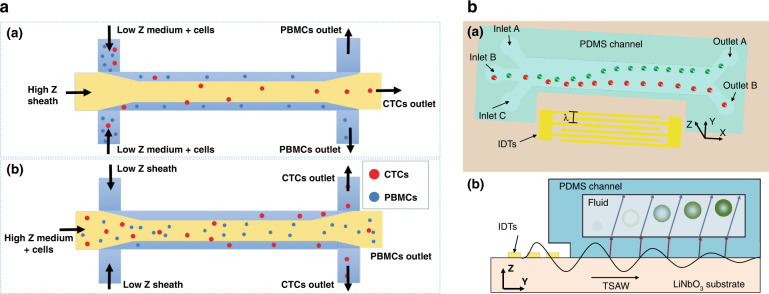
Acoustofluidic separation based on nonsize properties. **a** Separation of HeLa and MDA-MB-231 cells from peripheral blood mononuclear cells based on the acoustic impedance difference. Reproduced from ref. ^71^ with permission from the Royal Society of Chemistry. **b** Separation of polystyrene and polymethyl methacrylate particles with the same diameters based on the differences in particle density and propagation speed of sound using a traveling surface acoustic wave device. Reproduced from ref. ^73^ with permission from the American Chemistry Society.

Although size-based separation methods have shown great success in separating cells or particles of different sizes, they cannot separate particles of the same size or particles with small size differences. In such cases, separation methods based on other physical properties have been explored^69^. The acoustic impedance (*Z*), which is related to the density and speed of sound through the material, has been selected as a candidate property for separating particles or cells. It has been demonstrated that when certain conditions are met, the migration direction of the particles can be determined by the impedance difference of the particle and the medium^70^. If the acoustic impedance of particles is higher than that of the medium (*Z*_*p*_ > *Z*_*m*_), the particles migrate toward the pressure node. Similarly, the particles migrate toward the pressure antinode if the impedance is lower than that of the medium (*Z*_*p*_ < *Z*_*m*_). If there is no impedance difference between the particle and the medium, the particles encounter only acoustic streaming. Karthick et al. successfully developed such an acoustic impedance-based separation device to isolate circulating tumor cells (CTCs) from PBMCs in 1 h with >86% recovery and >50-fold enrichment (Fig. 4a)^71^. By adjusting the impedances of the middle medium and sheath liquid compared to those of different cells, both the high-impedance CTCs (i.e., HeLa) and the low-impedance CTCs (i.e., MDA-MB-231) could be separated from PBMCs. One limitation of this acoustic impedance-based method is that it requires adjusting the medium’s impedance levels, which may be harmful for live cells. Thus, separation methods that rely solely on particle properties have been investigated. Jo et al. developed a sheathless device to separate particles of the same size but different densities^72^. In this study, two pairs of IDTs were used to form SSAWs in the channel but configured with different pressure node positions. All particles would aggregate in the center pressure node when particles passed the first IDT pair due to the relatively long-term exposure to ARF. When particles passed the second IDT pair area where the pressure nodes were configured to the sidewalls, the high-density particles migrated toward the sidewalls further than the low-density particles due to the relatively short-time exposure to ARF, resulting in particle separation. Ma et al. proposed a method to separate particles based on the difference in density and speed of sound through the particles using a TSAW device (Fig. 4b)^73^. They theoretically and experimentally demonstrated that PS and polymethyl methacrylate (PMMA) particles with the same diameters exhibited nonlinear and distinct acoustophoretic responses as a function of their density, the speed of sound through them, and the applied TSAW frequency. Recently, Liu et al. demonstrated the taSSAW-based separation of SiO2, PMMA and PS particles, which have the same volume and different densities^74^. The team optimized the working parameters, such as the peak-to-peak voltage of the IDTs, the maximum flow velocity, and the fork-optimal flow ratio coefficients, using simulations to achieve precise sorting. The experimental results showed that the separation rates and purities were all above 90% for the three particles.

## Biological applications of acoustofluidic separation technologies

Acoustofluidic separation has a wide range of biological applications, including micron-sized cells and submicron bioparticles. Table 2 lists some representative applications.

**Table 2:** Representative biological applications of acoustofluidic separation.

Separated bioparticles	Acoustic types	Results	Advantages	Ref.
CTCs	taSSAW	1.2 ml/h flow rate; >87% recovery rate of MCF-7 and HeLa cells	High throughput	^76^
	BAW	Isolation of 4T1 cancer cells from whole blood samples with 96% efficiency; completely simulate the clinical treatment procedure	Cost-efficient; easy operation; high efficiency	^82^
	BAW	Isolation of A549 lung cancer cells with 100% purity, 92% separation efficiency in 15 minutes	High purity; high efficiency; fast processing	^81^
Bacteria	taSSAW	The separation of *E. coli* from RBCs with more than 96% purity	High purity; easy operation	^84^
	SSAW	*E. coli* was separated from human RBCs with 92.7% purity and >25 mm/s flow rate	High throughput and rapid separation	^85^
	BAW	~97% isolation efficiency of *S. aureus*	High resolution; high efficiency	^88^
Blood cells	BAW	RBCs and WBCs removal and platelet recovery of 80% using 10 mL/min flow rate	Vertical acoustic force was used for separation	^91^
	BAW	31.8% plasma yield and 99.9% plasma purity; ~5.8 μL/min; realization of ~17 pg/μL of target HIV p24 antibodies from whole blood sample	Microstreaming can be used as a micropump for the sample; perfect integration with downstream components	^89^
	SSAW	99% RBCs and WBCs removal ratio; nearly 98% platelet purity	High purity; whole blood separation	^90^
Viruses	BAW	The yield of MS2 was >90% and 80% of *saccharomyces cerevisiae* were removed	Good separation performance; early proof-of-concept device based on BAWs	^93^
	BAW	The separation purities of Dengue viruses and human lymphocytes are 98% and 70%, respectively	Novel structure design	^94^
	TSAW	The JEV virus was separated from the complex biological samples	Easy to operate; mild to target virus	^95^
Proteins	BAW	The separation efficiency of blood cells >90%; the releasing efficiency of streptavidin >75%	Bioaffinity microbead-assisted methods	^98^
	TSAW	The human thrombin, IgE, and mCardinal2 protein were successfully separated	Triseparation of proteins simultaneously	^99^
Exosomes	SSAW	>90% isolation yield of exosomes	One-step direct isolation of exosomes	^101^
	taSSAW	Isolation of exosomes from whole blood with 99.999% blood cells removed	Excellent sorting efficiency of exosomes; whole blood separation;	^102^

### Separation of cells

#### Separation of cancer cells

**Fig. 5:**
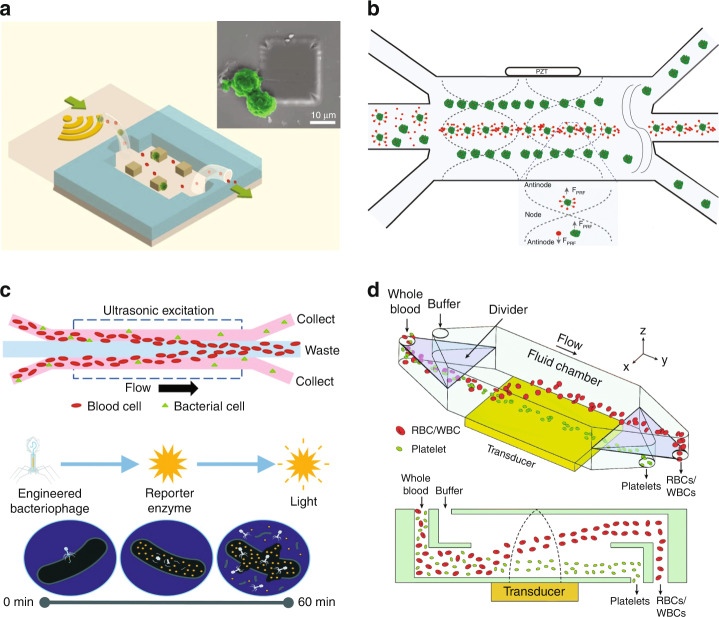
Acoustofluidic separation of cells. **a** An acoustic microfluidic trap array to separate cancer cells. Reproduced from ref. ^77^ with permission from Wiley Online Library. **b** A microBubble-Activated Acoustic Cell Sorting (BAACS) method to separate HCT 116 colon cancer cells. Reproduced from ref. ^80^ with permission from SpringerLink. **c** Bacterial separation from red blood cells based on different acoustophoretic responses using a low-cost plastic bulk acoustic wave-based device. Reproduced from ref. ^87^ with permission from the Royal Society of Chemistry. **d** High-throughput separation of red blood cells/white blood cells and platelets from whole blood using a vertical acoustic force. Reproduced from ref. ^91^ with permission from the Royal Society of Chemistry.

Cancer remains a major disease burden worldwide. In-depth studies of cancer cells not only contribute to the understanding of the mechanisms of cancer development and progression but also facilitate the development of medical interventions. Precise and efficient isolation of cancer cells from normal cells is an important prerequisite for cancer research. Both SSAW- and TSAW-based devices have been developed for the isolation of cancer cells^69,75^. Li et al. developed a taSSAW-based chip for the isolation of CTCs from white blood cells (WBCs)^76^. To significantly improve the separation throughput, which is critical for the practical application of CTCs, the authors designed both numerical and experimental models to systematically optimize multiple design parameters, including tilt angle, flow rate, IDT length, and input power. The optimized device was shown to be capable of isolating rare CTCs from WBCs at a flow rate of 1.2 ml/h. The recovery rate was validated to be >87% for MCF-7 and HeLa cells and >83% for four other cancer cell lines. Lu et al. used acoustic microstreaming traps to separate MCF-7 breast cancer cells (Fig. 5a)^77^. An array of micropillars were patterned inside the microchannel. When the SAWs were generated, microstreaming formed around the micropillars. Cancer cells were captured by the nearest pillar trap due to their large size, whereas other cells flowed out with the fluid. After the acoustic vibration stopped, the captured cancer cells could be released. Using this device, it is possible to achieve a capture efficiency of 95% for MCF-7 in spiked saline buffer and diluted serum and 66% for the whole blood samples. Furthermore, the platform is compatible with affinity-based sorting methods, thus showing great potential to further improve the isolation efficiency and specificity for clinical detection and diagnosis.

Cancer cell isolation has also been investigated in BAW-based devices^78,79^. Faridi et al. developed a microfluidic-based microBubble-Activated Acoustic Cell Sorting (BAACS) system to sort HCT 116 colon cells; the system relies on the specific binding of cancer cells to the surface-functionalized microbubbles and the different acoustic contrast coefficients of microbubbles and cells in the acoustic field (Fig. 5b)^80^. Due to the strong negative acoustic contrast coefficient of the microbubbles, the target cells bound to the microbubble moved to the pressure antinodes, whereas the unbound cells migrated to the pressure nodes due to their positive acoustic contrast coefficient. More than 75% sorting efficiency was achieved using this device. Iranmanesh et al. developed an acoustofluidic device that combines the separation and trapping of A549 lung cancer cells from RBCs in a single chip^81^. This device consisted of a prealignment zone, a size-based separation zone, and a trapping zone, which were realized by placing three ultrasound transducers with different frequencies (4.4 MHz, 1.39 MHz, and 2.78 MHz) consecutively along the channel. Cancer cells were enriched 130-fold with 100% purity, 92% separation efficiency, and 93% trapping efficiency in a 15-minute continuous process. Bai et al. reported acoustic microstreaming-induced isolation of 4T1 cells from whole blood samples of postoperative mice^82^. With the piezoelectric transducer and PDMS channel bonded on the glass slide, acoustic waves were transmitted into the channel, which consisted of an array of microcavities. The team optimized the geometry of the microcavities so that the velocity of the acoustic energy-induced fluid in the microcavities was higher than that in the main channel. As a result, when the acoustic waves were generated, larger CTCs were captured in the cavities and separated from other substances in the blood. This device demonstrated a high capture and separation efficiency (>96%).

#### Separation of bacteria

Bacterial infection can sometimes cause severe symptoms. Rapid and effective isolation of bacteria from complex body fluids can help identify the type of bacteria and confirm the diagnosis. Although bacteria are usually irregular in shape and smaller than eukaryotic cells, they can still be affected by acoustic waves and separated accordingly. Ai et al. isolated *Escherichia coli* (*E. coli*) from PBMCs using a standard SSAW device^83^. In this device, PBMCs were shifted to the pressure nodes on the sidewalls due to their larger size, whereas *E. coli* were concentrated in the center of microchannel by sheath flow with a purity of 95.65%. Li et al. used a taSSAW device to separate *E. coli* from RBCs with more than 96% purity^84^. When using whole blood samples, their device was able to remove RBCs and WBCs but failed to separate *E. coli* from platelets. Nevertheless, the authors demonstrated that this enrichment could decrease the nonspecific signals during the downstream electrochemical detection of *E. coli* in whole blood samples. Ning et al. designed a serpentine acoustofluidic device for the separation of *E. coli* bacteria from WBCs^85^. Unlike the previously mentioned serpentine channel for particle concentration^75,86^, this device placed samples in an acoustic field to extend the action length; thus, a higher flow rate could be used. For successful and accurate separation, the width and spacing of the channel were integer multiples of half wavelengths. Larger WBCs migrated to the pressure nodes, whereas the smaller bacteria remained in the middle of the channel due to the weaker ARF, causing them to flow out from different outlets. The result showed 92.7% separation purity and a 25 mm s^−1^ flow rate, which is higher than similar devices reported previously.

In addition to SAW-based devices, BAW-based devices have also been used for bacterial isolation. Dow et al. developed a low-cost plastic BAW device to isolate three pathogenic bacteria, including *Pseudomonas aeruginosa*, *Staphylococcus aureus (S. aureus)*, and *E. coli*, from RBCs or whole blood at clinically relevant concentrations^87^. The mixture sample was input into the chip from both side inlets. The blood cells were shifted to the center outlet due to the larger acoustophoretic response, while the bacteria were less affected by the ARF and collected from the side outlets (Fig. 5c). By incorporating a bacteriophage-based luminescence assay after acoustophoretic separation, a 33-fold improvement in the detection limit was demonstrated compared with the unpurified sample. Assche et al. presented an interesting method for the isolation of *S. aureus* from blood lysate called gradient acoustic focusing^88^. Instead of using the whole blood sample directly, the blood sample was preprocessed with lysis agent. The large cells and platelets were lysed without damaging the viability of the target bacteria. The theoretical and experimental studies confirmed that when the acoustic impedance of the central liquid (Z1) is larger than that of the particle suspension (Z0), the BAW-driven ARF suppressed acoustic streaming, and forced particles migrate to the central position of the channel from the sidewalls. As a result, the isolation of *S. aureus* achieved a high efficiency of 97.0 ± 0.9%. Moreover, this technique could separate submicron particles and cells, showing the ability for applications of other substances.

#### Separation of blood cells

Blood serves as a circulating carrier that provides the body with various nutrients and oxygen and removes waste. The numerous cells in the blood characterize the physiological state of the body. Changes in the number and state of blood cells often lead to diseases. The isolation of specific cells from blood can help to diagnose and treat health problems. Petersson et al. demonstrated the separation of RBCs and platelets using a BAW-based device^59^. Cells were added through the side inlets. Cesium chloride solution was added through the middle inlet to manipulate the relative density between the cells and fluid to enhance the separation efficiency. However, whether the added solution has an irreversible negative impact on the cells needs to be further studied. Cells were separated and flowed out of different outlets based on their sizes and densities. The separation of RBCs, platelets, and leukocytes in the buffy coat was also investigated using this device. While the efficiency of separating multiple cells simultaneously was not high, this early BAW-based microfluidic device was instrumental in promoting the use of similar systems in blood cell isolation. Liu et al. demonstrated that bubble-driven acoustic microstreaming can be used to separate cells and plasma in whole blood samples with a plasma purity of 99.9%^89^. Rectangular lateral cavities were positioned at a 15° angle from the channel to trap air bubbles. An acoustic microstreaming vortex was generated by the trapped bubbles with the vibration of the piezoelectric substrate. The size- and density-dependent trapping principle contained the larger blood cells in the vortex, allowing the blood plasma to flow downstream. Meanwhile, the fluid vortex acted as a micropump, which facilitated the continuous flow of fluid without integrating other pump equipment. Furthermore, the device utilized a similar bubble-driven microstreaming effect to enhance the mixing of the antigen-conjugated PS beads and HIV-p24 antibodies in plasma and trap the complexes for fluorescent detection to assess the concentration of HIV-p24 antibodies. This multifunctional device shows its ability to rapidly separate and detect biomarkers in blood samples.

With the development of acoustofluidic theory, SAWs have also been applied for the separation of cells in blood. Nam et al. separated platelets from undiluted whole blood using a classic SSAW device^90^. Sheath flows were input from side inlets to hydrodynamically focus the blood sample (0.25 µl/min) in the middle of the channel. Actuated by SSAW, large cells (RBCs and WBCs) migrated to sidewalls where pressure nodes formed, and platelets were extracted from the middle outlet due to their smaller diameter. The 99% RBC and WBC removal ratio and nearly 98% platelet purity demonstrated the good performance of this method. Chen et al. also developed an acoustic microfluidic device for platelet separation from whole blood with higher throughput^91^. Instead of using lateral cell migration, the vertical acoustic force was harnessed for separation. RBCs/WBCs were subjected to a stronger vertical acoustic force and were pushed to the upper layer, while the platelets were kept in the bottom layer (Fig. 5d). They achieved RBC/WBC removal and platelet recovery of 80% using a 10 mL/min flow rate and slightly higher efficiency when using a 5 mL/min flow rate. The balance between high throughput and sorting efficiency requires further investigation for this application.

### Separation of bionanoparticles

#### Separation of viruses

**Fig. 6:**
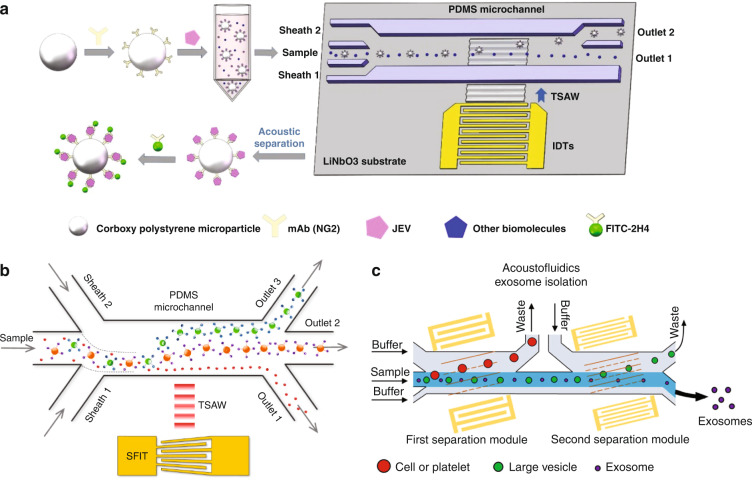
Acoustofluidic separation of bionanoparticles. **a** Separation of Japanese encephalitis virus from complex biological samples. Reproduced from ref. ^95^ with permission from Elsevier. **b** Triseparation of proteins from the mixture based on aptamer-coated microparticles and TSAW. Reproduced from ref. ^99^ with permission from the American Chemistry Society. **c** Exosome separation from plasma samples using a multistage acoustofluidic device. Reproduced from ref. ^104^ with permission from Nature.

Viruses are small particles of genetic material that are surrounded by a protein coat. The size of most viruses varies in diameter from 20 to 400 nm. Viruses can infect host cells and cause various diseases, such as AIDS, hepatitis, and COVID-19. Currently, polymerase chain reaction (PCR) and enzyme-linked immunosorbent assays (ELISAs) are considered the gold standard for virus detection. Both methods are capable of detecting viruses accurately. However, a long detection time, a requirement for complicated equipment, and the need for professional operation remain the common deficiencies of these methods^92^. Therefore, new strategies are needed for isolating and detecting viruses rapidly and accurately. The application of acoustofluidic devices in virus isolation has been investigated. Since the size of the virus is too small to be affected by ARFs, viruses have been concentrated by removing the cells using ARF from the virus–cell mixture. Jung et al. demonstrated a microfluidic BAW-based device that can isolate *Saccharomyces cerevisiae* (*S. cerevisiae*) and MS2 bacteriophage^93^. The sample mixture and deionized water were input into the two inlets of the H-filter device. A standing wave was formed, and the pressure node was located at the center of the channel. The ARF drove the larger *S. cerevisiae* cells toward the pressure node, and then cells flowed out to a different outlet other than the unaffected MS2 bacteriophage. The results showed that yields of MS2 were greater than 90%, and 80% of the *S. cerevisiae* were removed. Similarly, with the application of BAWs, Fong et al. designed a unique channel structure for the separation of cell-free Dengue viruses (50 nm) from human lymphocytes (5–8 μm)^94^. A second fluid channel was fabricated parallel to the main channel, which formed a thin silicon wall called a “transparent wall” to decouple the fluid and acoustic boundaries. As a result, asymmetric pressure nodes can be generated in the fluidic channel, which can push the cells further into the other half-channel and achieve better separation. The results showed that the separation purities of Dengue viruses and human lymphocytes were 98 and 70%, respectively. Recently, the specific separation of Japanese encephalitis virus (JEV) from complex biological samples was realized by capturing the virus first using antibody-functionalized microparticles and then enriching the particle-virus composites using a TSAW device (Fig. 6a)^95^.

#### Separation of proteins

Proteins are a class of macromolecules that perform a diverse range of functions within organisms. The effective sorting and accumulation of proteins are central goals in protein biotechnology. Due to the small size of proteins, ARF is not sufficient to directly manipulate proteins in typical acoustofluidic setups. Instead of using a flow-based device, Neumann et al. applied SSAWs on supported lipid bilayers (SLBs) and demonstrated the redistribution of proteins on planar SLBs^96^. When SAWs were coupled with SLBs, the membrane density was modulated, resulting in lipid transport and accumulation. The proteins anchored to the SLB by binding approaches, such as biotin-avidin, hydrophobic, and electrostatic interactions, could also be patterned using the same setup. By tuning the two IDTs with slightly different frequencies, lipid and protein transport was realized due to the shifting of the SAW pattern. Furthermore, protein separation was observed when two different proteins with different properties, such as molecular weight, isoelectric point, and ability to crystallize, were deposited on the same SLB, which was attributed to their competition for the antinode position. For example, separation of streptavidin and avidin was demonstrated despite their similarity in size and nature but differences in their ability to crystallize. In addition to the direct acoustic separation of proteins on SLBs, bioaffinity microbead-assisted methods have been adopted to separate proteins. For instance, Ahmad et al. successfully separated thrombin from mCardinal2 and human serum samples by capturing the proteins using aptamer-functionalized PS beads in a TSAW device^97^. Similarly, Li et al. developed a type of thermally responsive polypeptide fused to ligands that acts as a linking agent to selectively capture proteins into silicone microparticles for separating specific proteins from blood using a BAW device^98^. In particular, the separated proteins could be released from the microparticles for downstream analysis by cooling the solution below the solubility temperature of the polypeptides. Using streptavidin spiked in blood plasma as a model protein, the authors demonstrated that their method could achieve a separation efficiency exceeding 90% with a limit of detection of 0.75 nM and a release efficiency greater than 75%. Afzal et al. proposed an SIDT-generated TSAW device to separate three different types of proteins simultaneously based on the selective capture of proteins using aptamer-coated microparticles of different sizes (Fig. 6b)^99^. Thrombin and IgE proteins were captured by two types of PS microparticles coated with apt15 and aptD17.4, respectively, while mCardinal2 proteins were unbound. The mixture was injected into the microchannel, and the three proteins were separated by ARF based on the difference in size.

#### Separation of exosomes

Exosomes are small extracellular vesicles secreted by cells that contain many constituents of the parent cells, including DNA, RNA, and protein. They are present in various body fluids and are important media of intercellular communication. Exosomes have been recognized to be particularly useful in disease diagnosis and therapeutic applications^100^. Accordingly, exosome separation technology from complex biofluids has attracted increasing research interest. Unlike traditional ultracentrifugation and filtration separation methods that usually require multiple operation steps, the acoustofluidics-based method allows separation in a continuous manner with less sample loss and a lower potential for structural damage, offering a promising approach for exosome separation. Due to the small size of exosomes (40–160 nm), a high ARF is required to separate them in acoustofluidic devices. Lee et al. developed an SSAW nanofilter to isolate exosomes (<200 nm) from other larger extracellular microvesicles (MVs) in cell culture media and stored RBC products^101^. The cutoff size and the separation performance were optimized by adjusting parameters such as the channel design, acoustic transducer design, and flow rate. A >90% isolation yield of exosomes was achieved using the system. Wu et al. developed a method to isolate exosomes directly from whole blood using a multistage acoustofluidic device^102^. The device consisted of two pairs of tilted-angle IDTs to generate taSSAWs. The first microscale cell-removal module was used to isolate large blood components (e.g., RBCs, WBCs, and platelets), and the second exosome-isolation module could isolate exosomes from MV mixtures with a purity of 98.4%. A 99.999% blood cell removal rate was achieved when the two modules worked together. Later, the same group used a similar device to study the effect of liquid viscosity on exosome separation^103^. The results demonstrated that the movement of particles in high-viscosity fluid was lower than that in low-viscosity fluid. The device was applied to separate exosomes from saliva samples. Recently, Wang et al. applied this model to isolate exosomes from plasma samples collected from mice with well-characterized closed-head injuries (Fig. 6c)^104^. Subsequent analysis demonstrated increased exosome secretion following traumatic brain injury (TBI), which provides a potential approach for the rapid diagnosis of TBI.

## Conclusion and perspective

In this review, we summarized the theories and mechanisms of acoustofluidic separation technology. Most existing separation methods are based on differences in particle size. For particles of similar size, acoustofluidic separation methods based on other properties (such as impedance and density difference) have shown feasibility and great potential. The applications of these methods have been widely and successfully demonstrated in biological fields, including the separation of different types of cells and various bionanoparticles. While current technologies have the advantages of being contactless and biocompatible, relatively simple to operate, and having relatively high sorting efficiency, new challenges and opportunities coexist in future clinical practice, basic research, and commercialization.

Although the current acoustofluidic separation devices and platforms have shown great efficiency, many still require the use of pretreated samples, which complicates the overall process and sometimes affects sample quality. The separation of targets directly from raw samples such as whole blood can not only simplify the operation process but also establish a direct link to the relevant diseases^105^, thus advancing the clinical application of this technology. In vitro diagnosis (IVD) and point-of-care testing (POCT) are considered the main clinical applications of acoustic separation techniques. However, a complete solution that can handle sample preparation, target separation, and biomarker detection remains to be developed, and the all-acoustic platform holds great promise. For example, CTCs can be isolated directly from the patient’s whole blood sample using ARF, and then the CTCs can be lysed using the strong acoustic energy of the subsequent sonication module to expose DNA in the fluid. Meanwhile, the acoustic bubble microstreaming effect can be used to pump the fluid and enhance the sample-reagent mixing to facilitate the detection process. But, the throughput of current acoustofluidic devices is inadequate for the rapid processing of large amounts of samples, which is one of the basic requirements for clinical applications. Optimization of the microchannel structure to allow a higher flow rate and multiple-unit parallelization are potential solutions to this problem. However, excessive throughput sometimes leads to a decrease in separation efficiency, so it is important to strike a balance between sorting performance and throughput.

The current devices mainly focus on 2D (vertical or horizontal) separation, but 3D separation has not yet been well investigated. A possible strategy is to properly design a 3D IDT array that can focus 3D acoustic fields on the microfluidic channel, which should highly improve the precision and flexibility of particle manipulation, especially for the separation of multiple targets in complex samples. Moreover, with the development of acoustic metamaterials with unusual acoustic parameters (for example, negative refractive index)^106–108^, integrating these materials into microfluidic devices can manipulate and control sound waves in ways that are not possible in conventional materials, which can further improve the spatial resolution and accuracy of acoustic separation. In addition, there are still technical limitations in sorting bionanoparticles. Although sorting of submicron bioparticles can be achieved by removing the larger particles in the sample, this method cannot effectively remove smaller bioparticles. Therefore, the combination with other methods, such as the immunoaffinity method, has become a feasible technique for the specific sorting of bionanoparticles. Other novel strategies to improve the separation resolution of submicron bioparticles remain to be developed. Repeated experiments are often required to assess the performance of acoustofluidic separation, which is time-consuming and inconvenient. Some numerical simulation models can partially solve this problem^109,110^; however, discrepancies between simulations and experiments still exist. Recently, Talebjedi et al. integrated an artificial neural network (ANN) prediction platform and the multiobjective optimization approach to optimize the performance of acoustic separation^111^, showing the great potential of machine learning methods to aid in experimental design.

At present, only a few acoustofluidic technologies have been commercialized. However, these products are neither microfluidic chip-based (e.g., the Biosep cell retention device developed by Applikon for high-density perfusion processes) nor used for sorting processes (e.g., z-Movi by LUMICKS for cell avidity measurement). To the best of our knowledge, there are no commercial products for acoustofluidic separation based on microfluidic chips. The following reasons may explain this. First, the production cost is an important factor for successful commercialization. The costs of current piezoelectric-based BAW devices or IDT-based SAW devices are still too high for commercial use in disposable products. Using detachable acoustic actuation components is a feasible solution, but achieving the same level of efficiency as conventional chips is still challenging. Other strategies to reduce chip costs remain to be studied. Second, translating laboratory technology into practical instruments is still not an easy task. The operation of acoustofluidic separation requires the use of many auxiliary instruments, such as function generators, power amplifiers, and fluid control equipment. Although developments in electronic integrated circuit technology have made the high-degree integration of these components possible, the relatively specific and narrow application scenarios and thus limited market size are probably another main reason that prevents companies from investing in this technology. The development of a universal system that is compatible with different sorting chips may expand the application field and attract more business investment to develop commercialized products.
